# Integrating PLOR and SPAAC Click Chemistry for Efficient Site-Specific Fluorescent Labeling of RNA

**DOI:** 10.3390/ijms26062601

**Published:** 2025-03-13

**Authors:** Yanyan Xue, Xiao Si, Daxu Yin, Shengzhe Zhang, Hua Dai

**Affiliations:** 1 Institute of Translational Medicine, School of Medicine, Yangzhou University, Yangzhou 225001, China; six86951@163.com (X.S.); yyc19721222@163.com (D.Y.); zhangshengzhe@yzu.edu.cn (S.Z.); 2 The Key Laboratory of the Jiangsu Higher Education Institutions for Nucleic Acid & Cell Fate Regulation (Yangzhou University), Yangzhou 225001, China; 3 State Key Laboratory of Microbial Metabolism, School of Life Science and Biotechnology, Shanghai Jiao Tong University, Shanghai 200240, China

**Keywords:** RNA, fluorescent labeling, click chemistry, ePLOR

## Abstract

Precisely fluorescently labeling specific nucleotide sites of RNA is critical for gaining insights into the structure and function of RNA through multiple fluorescence detection techniques. The position-selective labeling of RNA (PLOR) method provides a promising strategy to achieve this, wherein the fluorophore-modified NTPs can be co-transcriptionally introduced to specific sites of nascent RNA by using T7 RNA polymerase (T7 RNAP). However, due to steric hindrance limitations, the efficiency of T7 RNAP in recognizing and incorporating large fluorophore-modified NTPs into RNA is far from satisfactory. To overcome this challenge, in this work, we developed an efficient PLOR variant (ePLOR) for the site-specific fluorescent labeling of RNA by integrating PLOR with a post-transcriptional SPAAC (strain-promoted azido-alkyne cycloaddition) click chemistry reaction. The efficiency of the SPAAC reaction occurring on RNA is nearly 100%. Consequently, ePLOR enables the precise fluorescent labeling of designated sites across various structural regions of SAM-VI riboswitch and adenine riboswitch RNA, with labeling and synthesis efficiencies that are 2–2.5 times higher than those of PLOR. The strategy developed in this work can be used for the efficient synthesis of a broader spectrum of long-strand RNAs with site-specific fluorescent labeling and greatly facilitate the detection of the structure and function of these RNAs.

## 1. Introduction

RNA, with its multiple biological roles and mechanisms, is the fundamental molecule of life. It not only encodes and transmits genetic information but also folds into complex three-dimensional structures to mediate various biological functions. Elucidating the structure and conformational dynamics of RNAs is crucial for understanding their functions. At present, most of the biophysical or biochemical methods used to investigate RNA structures and functions require the RNAs to be chemically modified or fluorescently labeled. For instance, introducing ^13^C/^15^N modifications into RNA alleviates the issue of spectral overlap in nuclear magnetic resonance (NMR) [1,2,3,4], while incorporating fluorophores Cy3/Cy5 into RNA enables in-depth analyses of RNA dynamic structures using single-molecule Förster resonance energy transfer (smFRET) technology [5,6,7,8]. Precisely labeling specific structural regions or nucleotide sites of RNA can significantly improve the detection resolution of these methods, thereby facilitating the capture of more intricate RNA conformational switches and intermediate states.

**Figure 1:**
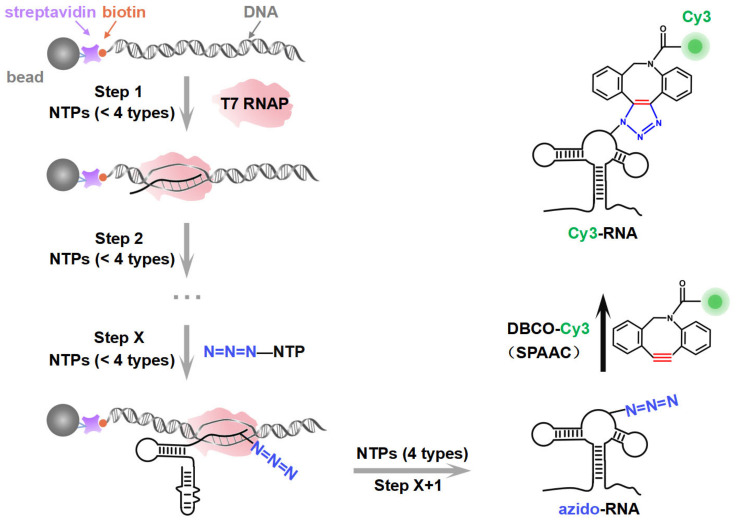
A schematic illustration of the synthesis strategy. The site-specific azido-labeled RNA (azido-RNA) is generated by means of PLOR through hybrid-phase transcription with a pause–restart mode (following the gray arrows). The transcription is divided into X + 1 steps, with azido-NTP incorporated into a specific position of the RNA at step X. The pause and restart at each step are precisely controlled by additions of limited NTPs to the solid phase. After step X + 1, the full-length azido-RNA is collected from the liquid phase. After purification, a post-transcriptional SPAAC click chemistry reaction between azido-RNA and DBCO-Cy3 is conducted to produce Cy3-RNA.

PLOR has been developed for the synthesis of site-specific labeled long-strand RNA [9,10,11]. PLOR employs a unique hybrid solid–liquid-phase transcription mode developed upon in vitro transcription, where the DNA template is attached to agarose beads as the solid phase, and transcription is initiated by adding T7 RNAP and NTPs as the liquid phase, thereby forming a DNA-RNA-T7 RNAP ternary transcription elongation complex (Figure 1, left). Since only three or fewer types of NTPs are added, T7 RNAP is compelled to halt transcription at specific sites due to the absence of the corresponding NTP type. The residual NTPs from the previous reaction step are then removed by solid-phase extraction (SPE), in which the ternary complex immobilized on the beads is rinsed through low-speed centrifugation (<200 g) to avoid cross-contamination. In subsequent elongation cycles, new NTP combinations (still limited to three or fewer types) are reintroduced into the solid phase to resume the transcription. Consequently, through multiple cycles of transcriptional pause and resumption, PLOR can strategically, co-transcriptionally incorporate modified NTPs into designated positions of the nascent RNA according to the designed scheme. Currently, various modifications, including ^13^C/^15^N isotopes; biotin; fluorophores Cy3, TQ3, Cy5, and 2-aminopurine; and heavy atoms Br, I, etc., can be introduced into specific sites of target RNAs through PLOR, greatly advancing structure and function studies of these RNAs [9,10,11,12,13,14]. However, as PLOR is a transcription system mediated by T7 RNAP, its efficiency in introducing NTPs with different modifications into RNA is constrained by T7 RNAP’s capacity to recognize the modified NTPs. While unmodified and small-molecule chemically modified NTPs (such as ^13^C/^15^N-NTP, Br, I, and 2-aminopurine) can be efficiently introduced into nascent RNA, the incorporation efficiency of large-fluorophore labeled NTPs (such as Cy3/Cy5-NTP) is significantly reduced due to steric hindrance. This limitation impairs the efficient synthesis of site-specific fluorescently labeled RNAs.

**Figure 2:**
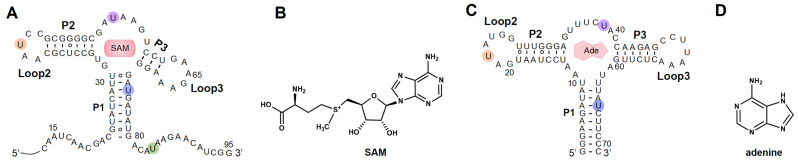
The secondary structures of RNAs synthesized by means of ePLOR. (**A**) The secondary structure of riboSAM. The labeled sites U43, U55, U74, and U84 are highlighted in orange, purple, blue, and green, respectively. (**B**) The chemical structure of SAM (the specific ligand for riboSAM). (**C**) The secondary structure of riboA. The labeled sites U22, U39, and U65 are highlighted in orange, purple and blue, respectively. (**D**) The chemical structure of adenine (the specific ligand for riboA).

In this study, by introducing azide functional groups, which are crucial in click chemistry, into designated sites of RNA, we report an efficient PLOR method, ePLOR, for fluorescently labeling specific sites of long-strand RNA based on a SPAAC click chemistry reaction (Figure 1). In recent years, the SPAAC “click” reaction, which does not require metal catalysts and occurs rapidly and efficiently under physiological conditions [15,16], has found increasing application in co-transcriptional RNA labeling [12,17,18]. The target RNAs synthesized in this study were the 95 nt SAM-VI riboswitch (riboSAM) and the 71 nt adenine riboswitch RNA (riboA) (the secondary structures of riboSAM and riboA are shown in Figure 2A,C) [9,12,13]. RiboSAM and riboA are riboswitch RNAs that can specifically recognize and bind their own ligands among numerous small-molecule metabolites in cells. This binding triggers structural changes that regulate the expression of downstream genes [19,20,21]. The ligands for riboSAM and riboA are S-adenosyl-methionine (SAM) and adenine, respectively, as shown in Figure 2B,D. Upon binding to their ligands, both riboSAM and riboA adopt a conformation featuring three stems (P1, P2, and P3) connected by a central three-way junction, with a ligand-binding pocket formed at the junction. In order to investigate whether ePLOR can efficiently label designated sites within arbitrary structural regions of target RNAs, azido-modified NTP was incorporated into specific positions within different structural regions of riboSAM and riboA, including stem-loops, internal loops, double helices, and single strands through distinct PLOR schemes (the labeling sites are shown in Figure 2A,C). This yielded azido-riboSAM/riboA. Subsequently, based on the SPAAC reaction between azide and the orthogonal reagent dibenzocyclooctyne (DBCO) reported in the literature [17,18], we explored the post-transcriptional SPAAC reaction system for azido-riboSAM/riboA and DBCO-Cy3/Cy5 (the chemical structures of azido-UTP and DBCO-Cy3/Cy5 are shown in Figure S1). We found that the SPAAC reaction occurring on different structural regions of riboSAM and riboA exhibited an efficiency approaching 100% in both instances, thereby facilitating efficient fluorescent labeling of specific sites on both RNAs using ePLOR. Furthermore, when compared to PLOR, ePLOR demonstrated a 2–2.5-fold increase in efficiency for synthesizing fluorescently labeled RNAs. This significant improvement addresses the inefficiencies associated with PLOR in fluorescent labeling, thus enabling the large-scale production of site-specific fluorescently labeled RNAs.

## 2. Results

### 2.1. Incorporation of Cy3 Fluorophore into Specific Sites of riboSAM Using ePLOR

**Figure 3:**
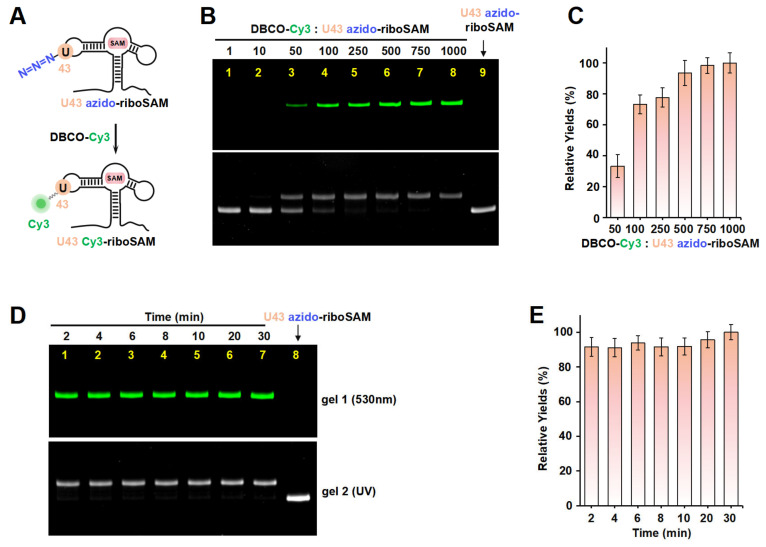
The incorporation of Cy3 into the U43 position on loop2 of riboSAM using ePLOR. (**A**) A diagram illustrating the reaction between U43 azido-riboSAM and DBCO-Cy3 to generate U43 Cy3-riboSAM. (**B**) PAGE images of the reaction products at different concentrations of DBCO-Cy3 under fluorescent (upper part) and UV (lower part) irradiation. U43 azido-riboSAM was loaded as a control. (**C**) Relative yields of U43 Cy3-riboSAM detected in (**B**). (**D**) PAGE images of the reaction products after different reaction times under fluorescent (upper part) and UV (lower part) irradiation. U43 azido-riboSAM was loaded as a control. (**E**) Relative yields of U43 Cy3-riboSAM detected in (**D**). Mean ± s.d. values of three replicates are shown.

Taking riboSAM as the target for labeling and synthesis, we first introduced an azide group into the U43 position within riboSAM’s loop2 using a seven-step PLOR reaction to obtain U43 azido-riboSAM (Figure 3A). All of the RNA and DNA sequences used in the PLOR are shown in Table S1, and diagrams of the PLOR generation of U43 azido-riboSAM are shown in Figure S2 and Table S2. Subsequently, we investigated the SPAAC reaction system between U43 azido-riboSAM and DBCO-Cy3. Given the DBCO content-dependent enhancement of reaction efficiency in SPAAC [22], we set up experiments with DBCO-Cy3 in molar amounts that were 1, 10, 50, 100, 250, 500, 750, and 1000 times that of U43 azido-riboSAM, and we used urea-PAGE to detect the production of U43 Cy3-riboSAM. Fluorescent gel imaging indicated that little U43 Cy3-riboSAM was generated in the presence of DBCO-Cy3 when the concentration of DBCO-Cy3 was below 50 times (i.e., 1 or 10 times). As the DBCO-Cy3 concentration further increased, the production of U43 Cy3-riboSAM gradually elevated, tending to reach the maximum reaction yield at 500 times and above (Figure 3B, upper part and Figure 3C). UV gel imaging displayed the same results (Figure 3B, lower part). Specifically, on the UV gel, U43 Cy3-riboSAM exhibited a shifted band in comparison to U43 azido-riboSAM due to the incorporation of the Cy3 group, which caused the U43 Cy3-riboSAM to migrate slightly slower. Furthermore, when the DBCO-Cy3 concentration reached 500 times or higher, virtually all of the U43 azido-riboSAM was converted to U43 Cy3-riboSAM. This demonstrated that the SPAAC reaction on RNA achieved an almost perfect reaction efficiency of approximately 100% (Figure 3B, lower part). After removing the excess DBCO-Cy3 through concentration and solvent exchange, we also used reverse-phase high-performance liquid chromatography (RP-HPLC) to characterize the U43 Cy3-riboSAM generated under a condition with a 1000-fold molar excess of DBCO-Cy3. In RP-HPLC, U43 Cy3-riboSAM eluted from the C8 column later than U43 azido-riboSAM (Figure S3), and was detectable upon fluorescence excitation at 550 nm. These observations were attributed to the hydrophobic and fluorescent properties of Cy3, further confirming the successful incorporation of Cy3 into the riboSAM. Moreover, under UV irradiation, the HPLC chromatogram revealed a single predominant peak corresponding to U43 Cy3-riboSAM, accompanied by only a minimal, nearly imperceptible peak attributable to U43 azido-riboSAM (Figure S3B). This observation strongly suggests that the SPAAC reaction proceeded with an efficiency approaching 100%, effectively converting virtually all of the U43 azido-riboSAM into U43 Cy3-riboSAM.

Under the conditions of the DBCO-Cy3 concentration being 500 times that of U43 azido-riboSAM, we further investigated the reaction rate. Urea-PAGE detection showed that the reaction between the two was extremely rapid, being essentially complete after 2 min. This was confirmed by the observation of equal amounts of U43 Cy3-riboSAM (fluorescent imaging) and the complete conversion of all U43 azido-riboSAM to U43 Cy3-riboSAM (UV imaging) at different reaction times (2, 4, 6, 8, 10, 20, and 30 min) (Figure 3D,E). These results demonstrate that the SPAAC reaction occurring on the riboSAM stem-loop was rapid and efficient, enabling the efficient incorporation of Cy3 into the U43 site.

**Figure 4:**
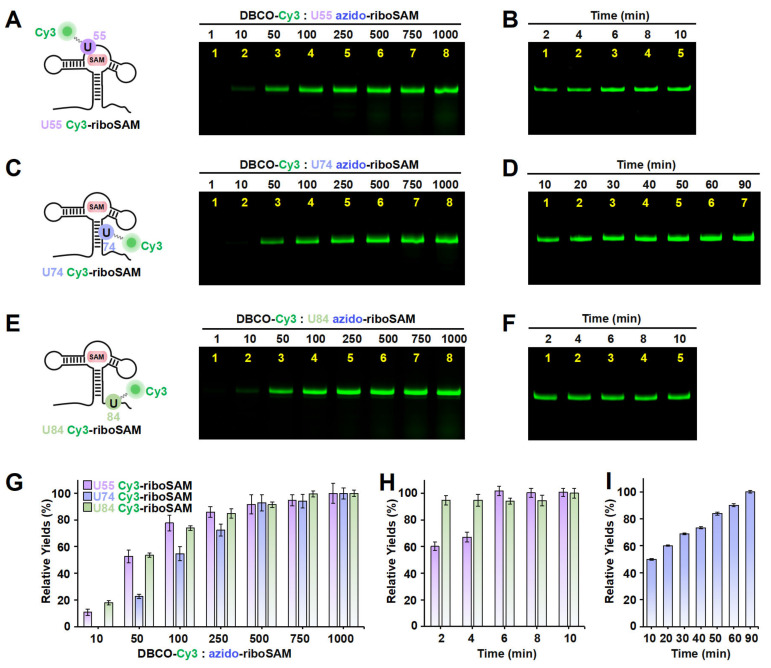
The incorporation of Cy3 into the U55, U74, and U84 positions of riboSAM using ePLOR. (**A**–**F**) Fluorescent PAGE images of U55 Cy3-riboSAM (**A**,**B**), U74 Cy3-riboSAM (**C**,**D**), and U84 Cy3-riboSAM (**E**,**F**) generated at different concentrations of DBCO-Cy3 (**A**,**C**,**E**) and after different reaction times (**B**,**D**,**F**). (**G**) Relative yields of the three RNAs detected in (**A**,**C**,**E**). (**H**) Relative yields of U55 Cy3-riboSAM and U84 Cy3-riboSAM detected in (**B**,**F**), respectively. (**I**) Relative yields of U74 Cy3-riboSAM detected in (**D**). Mean ± s.d. values of three replicates are shown.

Apart from the stem-loop, we sought to determine whether the SPAAC reaction on other structural regions of riboSAM would also be rapid and efficient. Next, we designed a new PLOR strategy to synthesize riboSAM with an azide group label at the U55 site of the internal loop (U55 azido-riboSAM) (Figure S4 and Table S3), and we similarly evaluated the yield of its reaction with DBCO-Cy3 to produce U55 Cy3-riboSAM (Figure 4A, left). Fluorescent gel imaging indicated that, similarly to that of U43, the production of U55 Cy3-riboSAM gradually increased as the DBCO-Cy3 concentration rose, reaching its maximum reaction yield at 500 times and above (Figure 4A,G). However, unlike the rapid reaction observed at U43, the reaction between U55 azido-riboSAM and DBCO-Cy3 required more than 6 min to complete (Figure 4B,H and Figure S5A,B). The crystal structure revealed that in the ligand-bound state, U35 of the truncated 55 nt riboSAM from the same family (corresponding to U55 in this study) is wrapped within the RNA interior (Figure S6) [23]. This suggests that the contact between U55 and DBCO-Cy3 may not be as direct and sufficient as that of U43, thus resulting in a slightly slower SPAAC reaction rate. We also examined the effect of the reaction temperature on the yield of SPAAC. Gel imaging indicated that the reaction efficiencies at 30 °C and 37 °C were significantly higher than that at 25 °C, especially at 37 °C (Figure S5C,D). Therefore, all SPAAC reactions in this study were conducted at 37 °C.

Furthermore, we conducted azide labeling at the U74 site (U74 azido-riboSAM) within the double-stranded region and the U84 site (U84 azido-riboSAM) within the single-stranded region of riboSAM (Figures S7 and S8 and Tables S4 and S5), and we detected the SPAAC reaction yields for U74/U84 azido-riboSAM (Figure 4C,E, left). Fluorescent gel imaging indicated that the production of U74/U84 Cy3-riboSAM also gradually increased as the DBCO-Cy3 concentration rose, reaching its maximum reaction efficiency when the DBCO-Cy3 concentration was 500–750 times higher than that of U74/U84 azido-riboSAM (Figure 4C,E,G). The reaction between U84 azido-riboSAM and DBCO-Cy3 was also completed within 2 min (Figure 4F,H). However, the difference lies in that the reaction rate between U74 azido-riboSAM and DBCO-Cy3 was significantly slower than that at other sites, with only half of the substrate reacting after 10 min and complete reaction not occurring until 90 min (Figure 4D,I). This may be due to the fact that U74 is located on a double helix and is buried inside the RNA molecule when riboSAM folds into a complex spatial structure, reducing its direct contact with DBCO-Cy3 compared to other sites (U43 on the stem-loop and U84 in a single-stranded region) (Figure S6). In summary, through ePLOR, we achieved efficient and rapid Cy3 labeling of specific sites in different structural regions of riboSAM.

### 2.2. Incorporation of Cy5 Fluorophore into Specific Sites of riboA Using ePLOR

**Figure 5:**
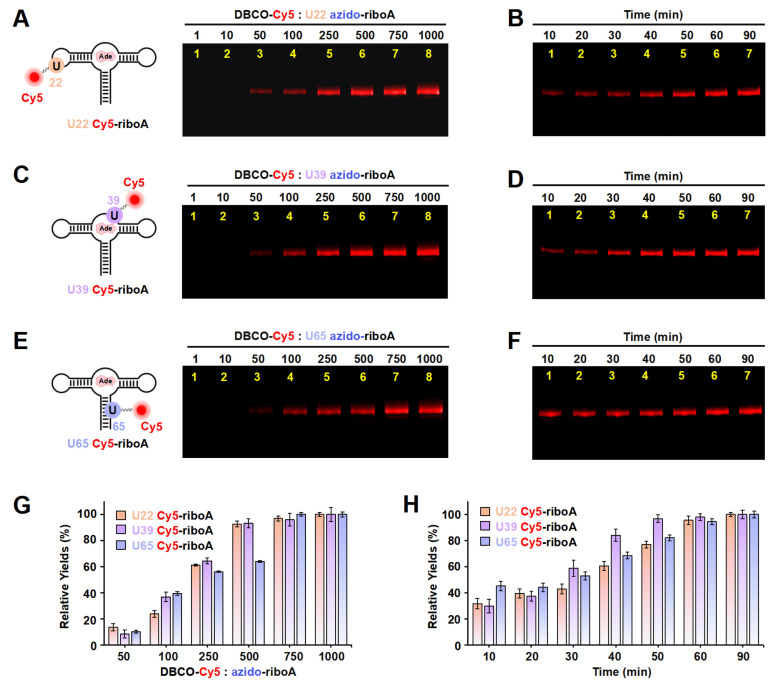
The incorporation of Cy5 into the U22, U39, and U65 positions of riboA using ePLOR. (**A**–**F**) Fluorescent PAGE images of U22 Cy5-riboA (**A**,**B**), U39 Cy5-riboA (**C**,**D**), and U65 Cy5-riboA (**E**,**F**) generated at different concentrations of DBCO-Cy5 (**A**,**C**,**E**) and after different reaction times (**B**,**D**,**F**). (**G**) Relative yields of the three RNAs detected in (**A**,**C**,**E**). (**H**) Relative yields of the three RNAs detected in (**B**,**D**,**F**). Mean ± s.d. values of three replicates are shown.

On the basis of the results above, in order to confirm the effectiveness of ePLOR in fluorescently labeling a wider array of target RNAs, we utilized ePLOR for fluorescent riboA synthesis. Similarly, we first designed distinct PLOR strategies to introduce azide groups into the U22, U39, and U65 sites within the stem-loop, internal loop, and double-stranded regions of riboA, respectively, obtaining U22 azido-riboA, U39 azido-riboA, and U65 azido-riboA (Figure S9 and Tables S6–S8). Additionally, we verified the SPAAC reaction between azido-riboA and DBCO-Cy5 to ascertain whether Cy5, like Cy3, could be efficiently introduced into designated sites of riboA. Urea-PAGE detection revealed that, as the DBCO-Cy5 concentration increased, the production of U22/U39/U65 Cy5-riboA also gradually increased, similarly tending to reach the maximum reaction yield when the DBCO-Cy5 concentration reached 500–750 times (Figure 5A,C,E,G). This indicates that, compared to the reactions observed on riboSAM, the SPAAC reactions conducted on riboA exhibit equally high efficiency, facilitating effective fluorescent labeling of different structural regions of riboA through the application of ePLOR. Additionally, we used RP-HPLC to characterize the U22 Cy5-riboA generated under a condition with a 1000-fold molar excess of DBCO-Cy5. Similar HPLC spectra were observed to those obtained for U43 Cy3-riboSAM, demonstrating that the SPAAC reaction in riboA also achieved an almost perfect reaction of approximately 100% (Figure S10).

We also detected the reaction rates that occurred on different positions of riboA. Notably, the SPAAC reactions observed at the stem-loop (U22) and internal loop (U39) of riboA were significantly slower than those at the corresponding sites on riboSAM. Specifically, the reactions at U22 and U39 were completed after approximately 60 min (compared to just 2 min for U43 on riboSAM) and 50 min (compared to 6 min for U55 on riboSAM), respectively (Figure 5B,D,H). The crystal structure revealed that U22 is located within the kissing loop formed by loop2 and loop3, extending toward the interior of the RNA molecule, while U39 is positioned within the ligand-binding pocket, also extending inward and forming a hydrogen bond with the ligand adenine (Figure S11) [9]. This leads to some spatial hindrance for direct contact between the azide groups at these two sites and DBCO-Cy5. Similarly, U65 on the double helix extends toward the interior of riboA, analogous to the U74 site on riboSAM’s double helix, and requires 60–90 min to react completely (Figure 5F,H).

### 2.3. Comparison of the Efficiency of PLOR and ePLOR for Fluorescent RNA Synthesis

**Figure 6:**
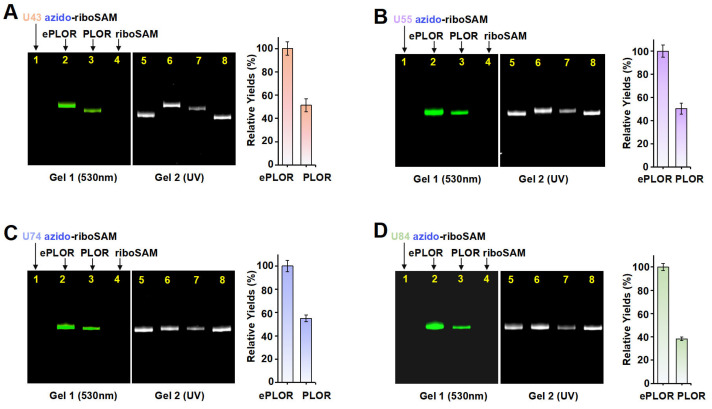
A comparison of efficiency between PLOR and ePLOR for site-specific Cy3-riboSAM synthesis. Fluorescent (**left**) and UV (**right**) PAGE images of U43 Cy3-riboSAM (**A**), U55 Cy3-riboSAM (**B**), U74 Cy3-riboSAM (**C**), and U84 Cy3-riboSAM (**D**) produced by means of ePLOR (lanes 2 and 6) and PLOR (lanes 3 and 7), respectively. The U43/U55/U74/U84 azido-riboSAMs (lanes 1 and 5) and unlabeled riboSAM (lanes 4 and 8) were loaded as controls. The relative yields from ePLOR were set to 100% in the individual RNA system.

To ascertain whether ePLOR can address the inefficiency of PLOR in the fluorophore labeling of RNA, we conducted a comparative analysis of the efficiency of PLOR and ePLOR in site-specific Cy3-riboSAM synthesis. By using PLOR to directly synthesize U43/U55/U74/U84 Cy3-riboSAM (Figures S2, S4, S7 and S8 and Tables S2–S5), we compared the efficiency of generating these four RNAs using PLOR and ePLOR. Urea-PAGE detection and a quantitative analysis showed that the yields of U43/U55/U74/U84 Cy3-riboSAM synthesized using ePLOR were consistently higher than those obtained with PLOR, being approximately 2–2.5 times greater (Figure 6A–D). This demonstrates that ePLOR indeed overcame the inefficiency of the PLOR method in fluorescent labeling and achieved the same efficiency for the site-specific introduction of fluorophores as for small-molecule chemical modifications or unmodified nucleotides. Specifically, when the total DNA amount in the PLOR system was considered 100%, the synthesis efficiencies of fluorescently modified U43/U55/U74/U84 Cy3-riboSAM via ePLOR were approximately 23%, 19%, 14%, and 13%, respectively. The excess DBCO-Cy3 molecules mixed in with the SPAAC reaction products can be efficiently removed through concentration and displacement using an ultrafiltration tube, with minimal impact on RNA loss during the process. Additionally, the filtered and recovered DBCO-Cy3 molecules can be reused for the next round of reaction.

## 3. Discussion

The precise fluorescent labeling of specified nucleotide sites on RNA is pivotal for the utilization of a wide range of fluorescent detection techniques in conducting comprehensive research into the structure and function of target RNA. In this work, by integrating the PLOR method with a post-transcriptional SPAAC reaction, we established an efficient PLOR variant, ePLOR, for the fluorescent labeling of specific sites with long-strand RNA. For ePLOR, the efficiency of the SPAAC reaction occurring on RNA was nearly 100%, effectively converting almost all PLOR-synthesized azido-RNA into Cy3- or Cy5-RNA. Consequently, the synthesis efficiency of site-specific fluorescently labeled RNA was enhanced by approximately 2–2.5 times. By using ePLOR, we achieved the efficient fluorescent labeling of designed sites within different structural regions of riboSAM and further validated this on riboA. This confirms that ePLOR can be applied to the efficient fluorescent labeling and synthesis of a broader spectrum of RNAs, with the labeled nucleotide positions not being affected by the RNA’s inherent structure. Further examinations revealed that, although the SPAAC reaction achieved nearly 100% efficiency across various structural regions, reactions occurring at exposed sites on the molecular surface, including single strands, stem-loops, and internal loops, proceeded more rapidly (2–6 min). In contrast, reactions at sites buried within double helices or the interior of the molecule were relatively slower, thus requiring longer reaction times (60–90 min). Currently, we are also attempting to apply ePLOR to the fluorescent labeling and synthesis of longer RNA molecules (greater than 200 nt) with crucial biological functions. This approach aims to facilitate the dynamic tracking of these RNAs both intracellularly and extracellularly, as well as enhancing the detection of their structures and functions.

## 4. Materials and Methods

### 4.1. Preparation of DNA Templates for PLOR Reactions

The DNA and RNA sequences used for the preparation of azido-riboSAM and azido-riboA are listed in Table S1. The biotin–DNA templates used in the PLOR reactions were prepared by means of PCR, with biotin and 2′-O-methyl groups at the 5′-ends of the coding and template strands, respectively. The biotin was used to attach the DNA templates onto streptavidin-coated agarose beads, enabling PLOR to easily separate the solid phase and exchange reagents and buffers via filtration. Additionally, the 2′-O-methyl modifications were employed to minimize the generation of undesired non-template transcripts. The biotin–DNA produced via PCR was purified by means of 10% urea-PAGE and then incubated with streptavidin-coated agarose beads (Smart-Life science, Changzhou, China) at 4 °C overnight to immobilize the DNA on the beads, producing solid-phase beads–DNA that served as templates for the PLOR reactions. These prepared immobilized beads–DNA were then kept at 4 °C for later use.

### 4.2. Preparation of Site-Specific Labeled riboSAM and riboA

PLOR was used to synthesize site-specific labeled riboSAM and riboA. The azido/Cy3-riboSAM samples, including U43 azido/Cy3-riboSAM, U55 azido/Cy3-riboSAM, U74 azido/Cy3-riboSAM, and U84 azido/Cy3-riboSAM, were prepared via the PLOR method, as described earlier [9,10,12]. The detailed synthesis procedure for the U43 azido/Cy3-riboSAM was divided into 7 steps. the reagents used at each step are listed in Table S2. In the first step, 1 mL of 10 µM beads–DNA and equal amounts of T7 RNAP, 1.12 mM ATP, 0.96 mM GTP, and 32 µM UTP in buffer (40 mM Tris-HCl, pH 8.0, 100 mM K_2_SO_4_, 6 mM MgSO_4_, and 10 mM DTT) were incubated at 37 °C for 15 min, generating the 12 nt riboSAM fragment. Filtration and bead-rinsing were then conducted at least three times using washing buffer (40 mM Tris-HCl, pH 8.0, 6 mM MgSO_4_) to remove the residual NTPs. Unless otherwise noted, filtration and bead-rinsing were conducted after each step. The transcription that proceeded in steps 2 to 7 was performed at 25 °C for 10 min in elongation buffer (40 mM Tris-HCl, pH 8.0, 6 mM MgSO_4_, and 10 mM DTT) with the addition of different NTP mixtures, as listed in Table S2. The azido-UTP (Jena Bioscience, Jena, Germany) and Cy3-UTP (APExBIO Technology, Houston, TX, USA) were introduced to the nascent riboSAM at steps 6A and 6B, respectively. A higher temperature of 30 °C was used in step 6B to improve the incorporation efficiency of T7 RNAP for the bulky Cy3. After azide and Cy3 incorporation, four types of NTPs were added to synthesize the full-length riboSAM, producing the U43 azido/Cy3-riboSAM. Detailed descriptions of the synthesis of U55 azido/Cy3-riboSAM, U74 azido/Cy3-riboSAM, U84 azido/Cy3-riboSAM, U22 azido-riboA, U39 azido-riboA, and U64 azido-riboA are provided in Tables S3–S8, respectively. The azido-labeled riboSAMs and riboAs were concentrated through centrifugation with an ultrafiltration tube and exchanged into DEPC-H_2_O for future usage.

### 4.3. Post-Transcriptional SPAAC Click Chemistry Reactions

The azido-riboSAMs were refolded in incubation buffer (40 mM Tris-HCl, pH 8.0, 100 mM K_2_SO_4_, 6 mM MgSO_4_) containing 0.5 mM SAM by heating them at 85 °C for 5 min and then cooling them down to room temperature before usage. The SPAAC click chemistry reactions between azido-riboSAMs and DBCO-Cy3 (Jena Bioscience, Jena, Germany) were performed in incubation buffer with 0.5 mM SAM, 0.5 µM azido-riboSAM, and 0.5–500 µM (0.5, 5, 25, 50, 125, 250, 375, and 500 µM, respectively) DBCO-Cy3 at 37 °C for 2–90 min. The reaction product (5 µL) and the corresponding purified azido-riboSAM were loaded onto 12% urea polyacrylamide analysis gel, and the gels were checked under UV irradiation or with a ChemiScope instrument (fluorescent excitation wavelength of 530 nm) (CLiNX, Shanghai, China). The experimental yields were detected by quantifying the gel band intensities using the ChemiScope instrument’s Gel analysis software (GenoSens Analysis 2000), and then dividing them by the amount of Cy3-riboSAM produced under the 500 µM DBCO-Cy3 condition. The azido-riboAs were reacted with DBCO-Cy5 (Jena Bioscience, Jena, Germany) under the same conditions, except that 1 mM adenine was added to the buffer instead of SAM [13]. The Cy5-riboAs were detected under fluorescence irradiation with a wavelength of 630 nm.

### 4.4. RP-HPLC Detection

U43 Cy3-riboSAM and U22 Cy5-riboA were detected by means of RP-HPLC using a C8 column (Phenomenex Luna, Torrance, CA, USA) via the following procedure: the first 2 min was with 10% buffer B (75% acetonitrile with 100 mM TEAA) and 90% buffer A (DEPC-H_2_O with 100 mM TEAA), which was then ramped from 10 to 70% buffer B over the next 50 min (with flow rates of 0.3 mL/min). The spectra were recorded under both UV (260 nm) and fluorescence (550 nm or 650 nm) irradiation.

### 4.5. Comparison of the Efficiency of PLOR and ePLOR

For this process, 0.5 mL of 10 µM PLOR reactions were conducted to produce azido-riboSAMs and corresponding Cy3-riboSAMs. The yields of azido-riboSAMs were detected via urea-PAGE, using the purified unlabeled riboSAM as a control. Further, the PLOR efficiency was obtained by dividing the yields by the amount of DNA templates used for generating the products. Then, 5 µL of azido-riboSAMs was reacted with DBCO-Cy3 at a DBCO-Cy3/RNA molar ratio of 1000 times. After the reaction had proceeded at 37 °C for 10–90 min, all the products were loaded onto the 12% urea polyacrylamide analysis gel, and 5 µL of the corresponding Cy3-riboSAM generated by means of PLOR was also loaded to compare the efficiency of these two methods. The azido-riboSAM and purified unlabeled riboSAM were also loaded as controls. The relative yields of ePLOR were set to 100% in the individual RNA system.

## Data Availability

The data presented in this study are available in the Supplementary Materials.

## Supplementary Materials

The following supporting information can be downloaded at https://www.mdpi.com/article/10.3390/ijms26062601/s1.

## Abbreviations

The following abbreviations are used in this manuscript:

PLOR	Position-selective labeling of RNA
T7 RNAP	T7 RNA polymerase
SPAAC	Strain-promoted azido-alkyne cycloaddition
ePLOR	Efficient PLOR
