# Supplementary material for: Integrating PLOR and SPAAC Click Chemistry for Efficient Site-Specific Fluorescent Labeling of RNA
Source: Int J Mol Sci. 2025 Mar 13;26(6):2601. doi: 10.3390/ijms26062601 (PMC11942227; doi:10.3390/ijms26062601)
Supplement: Supplementary file 1 [file ijms-26-02601-s001.zip › ijms-3503578-supplementary.pdf]

Supplementary Information for

Integrating PLOR and SPAAC Click Chemistry for Efficient Site-Specific Fluorescent Labeling of RNA

Yanyan Xue [1,2,3,*], Xiao Si [1], Daxu Yin [1], Shengzhe Zhang [1,2] and Hua Dai [1,2,*]

[1] Institute of Translational Medicine, School of Medicine, Yangzhou University, Yangzhou 225001, China; six86951@163.com (X.S.); yyc19721222@163.com (D.Y.); zhangshengzhe@yzu.edu.cn (S.Z.)

[2] The Key Laboratory of the Jiangsu Higher Education Institutions for Nucleic Acid & Cell Fate Regulation (Yangzhou University), Yangzhou 225001, China

[3] State Key Laboratory of Microbial Metabolism, School of Life Science and Biotechnology, Shanghai Jiao Tong University, Shanghai 200240, China

* Correspondence: yanyanxue@yzu.edu.cn (Y.X.); daihua@yzu.edu.cn (H.D.)

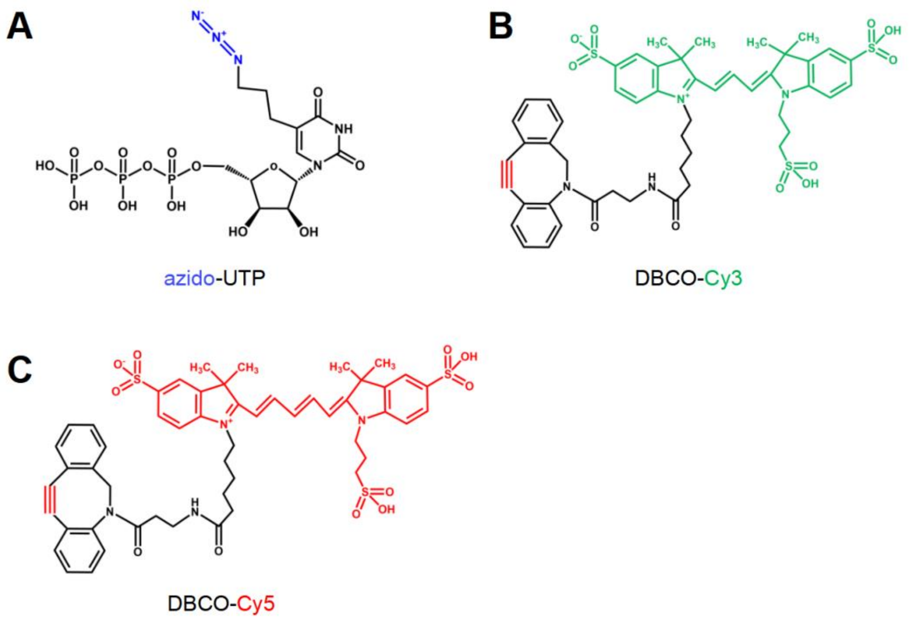

Supplementary Figure S1. The chemical structures of azido-UTP (A), DBCO-Cy3 (B) and DBCO-Cy5 (C).

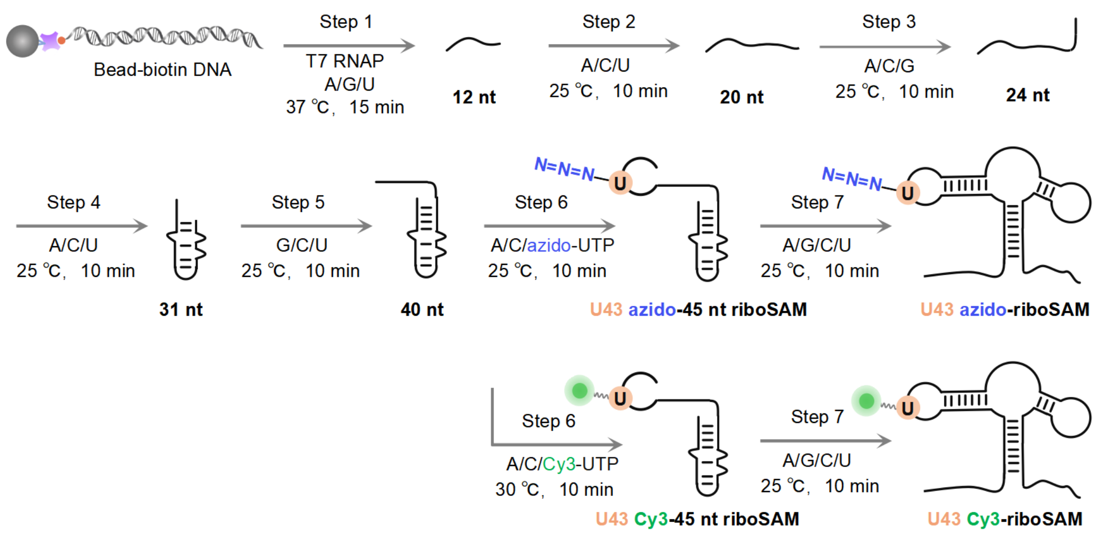

Supplementary Figure S2. The schematic procedures of 7-step PLOR for U43 azido-riboSAM and U43 Cy3-riboSAM synthesis.

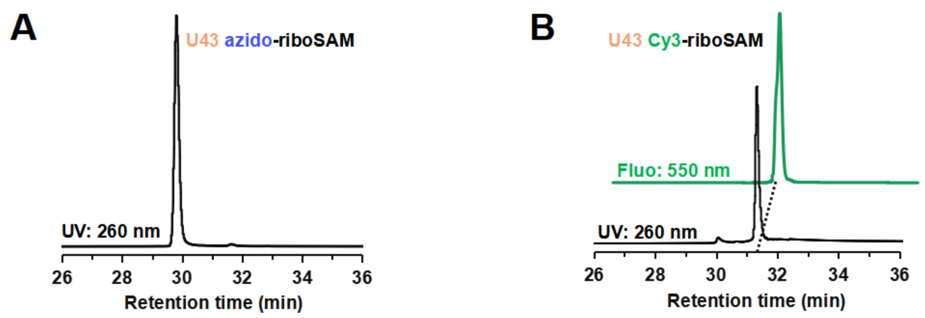

Supplementary Figure S3. Characterization of U43 Cy3-riboSAM via RP-HPLC. **(A)** HPLC spectrum of U43 azido-riboSAM at UV (260 nm) irridiation. **(B)** HPLC spectra of U43 Cy3-riboSAM under UV (260 nm, black line) and fluorescent (550 nm, green line) excitation.

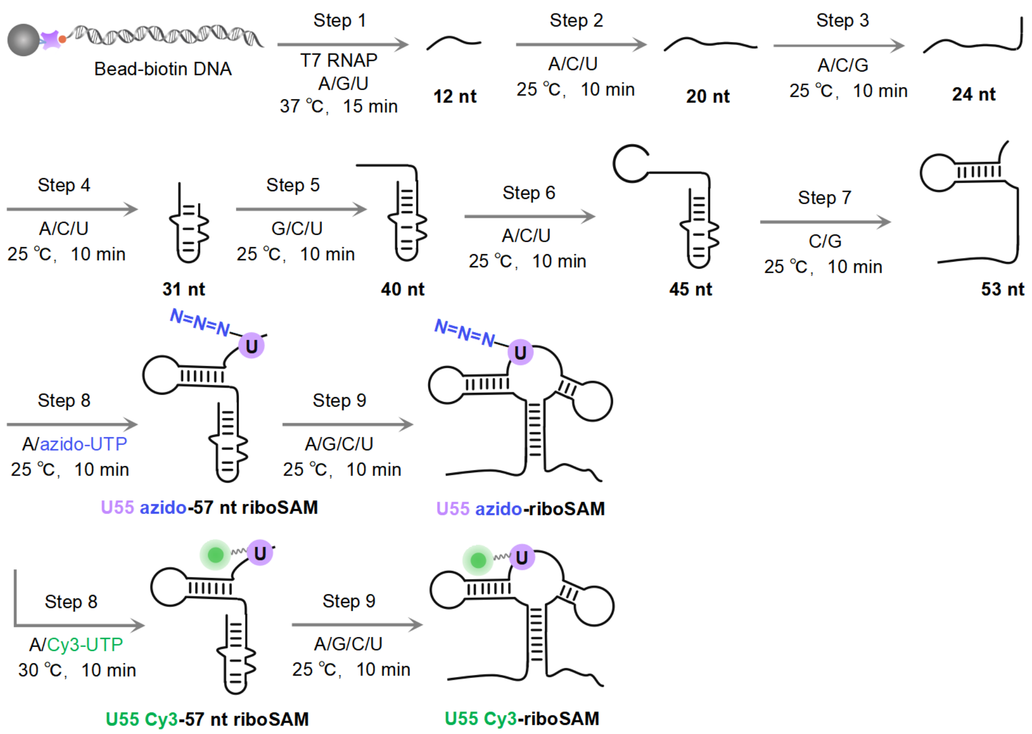

Supplementary Figure S4. The schematic procedures of 9-step PLOR for U55 azido-riboSAM and U55 Cy3-riboSAM synthesis.

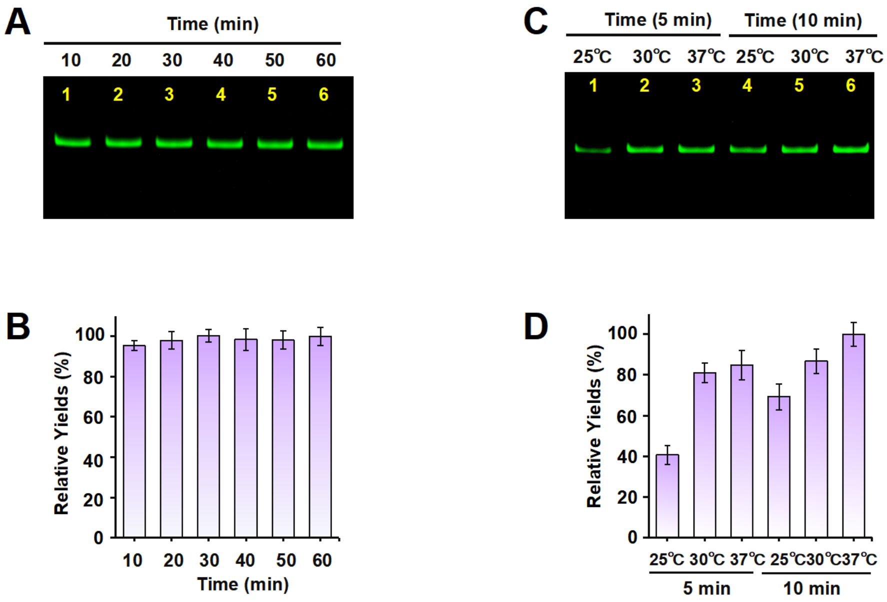

Supplementary Figure S5. Urea-PAGE detections of U55 Cy3-riboSAM generated at different times and temperatures. **(A, C)** Fluorescent PAGE images of U55 Cy3-riboSAM generated at different times (A) and temperatures (C). **(B, D)** Relative yields detected in (A) and (C), respectively. Mean ±s.d. values of three replicates are shown.

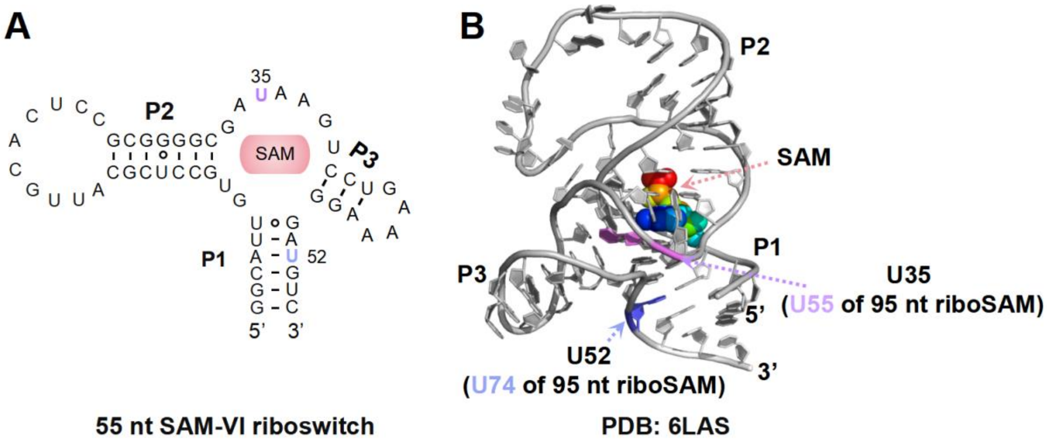

Supplementary Figure S6. The crystal structure of the truncated 55 nt riboSAM. **(A)** The secondary structure of riboSAM used to obtain the crystal structure shown in (B). **(B)** The crystal structure of riboSAM bound with SAM (PDB ID: 6LAS). U35 (corresponds to U55 in our study), U52 (corresponds to U74 in our study) are shown in purple and blue respectively. The ligand, SAM is shown as colorful balls.

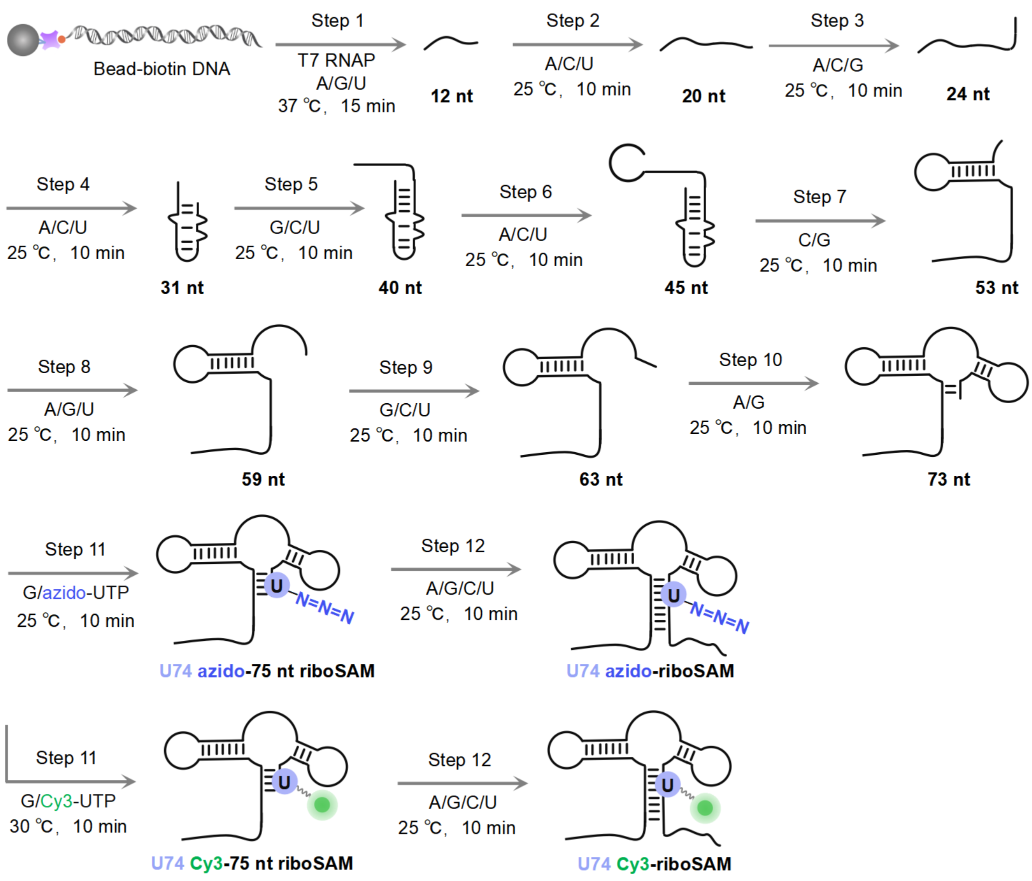

Supplementary Figure S7. The schematic procedures of 12-step PLOR for U74 azido-riboSAM and U74 Cy3-riboSAM synthesis.

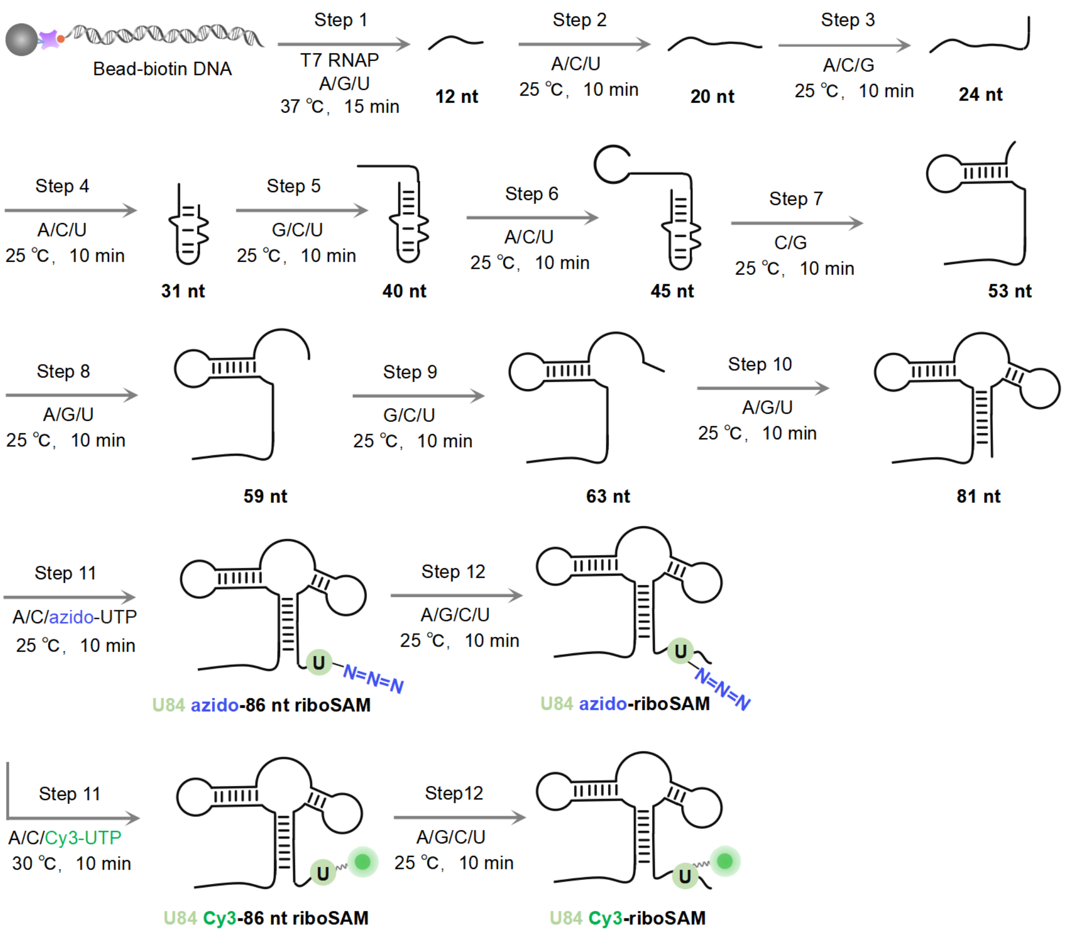

Supplementary Figure S8. The schematic procedures of 12-step PLOR for U84 azido-riboSAM and U84 Cy3-riboSAM synthesis.

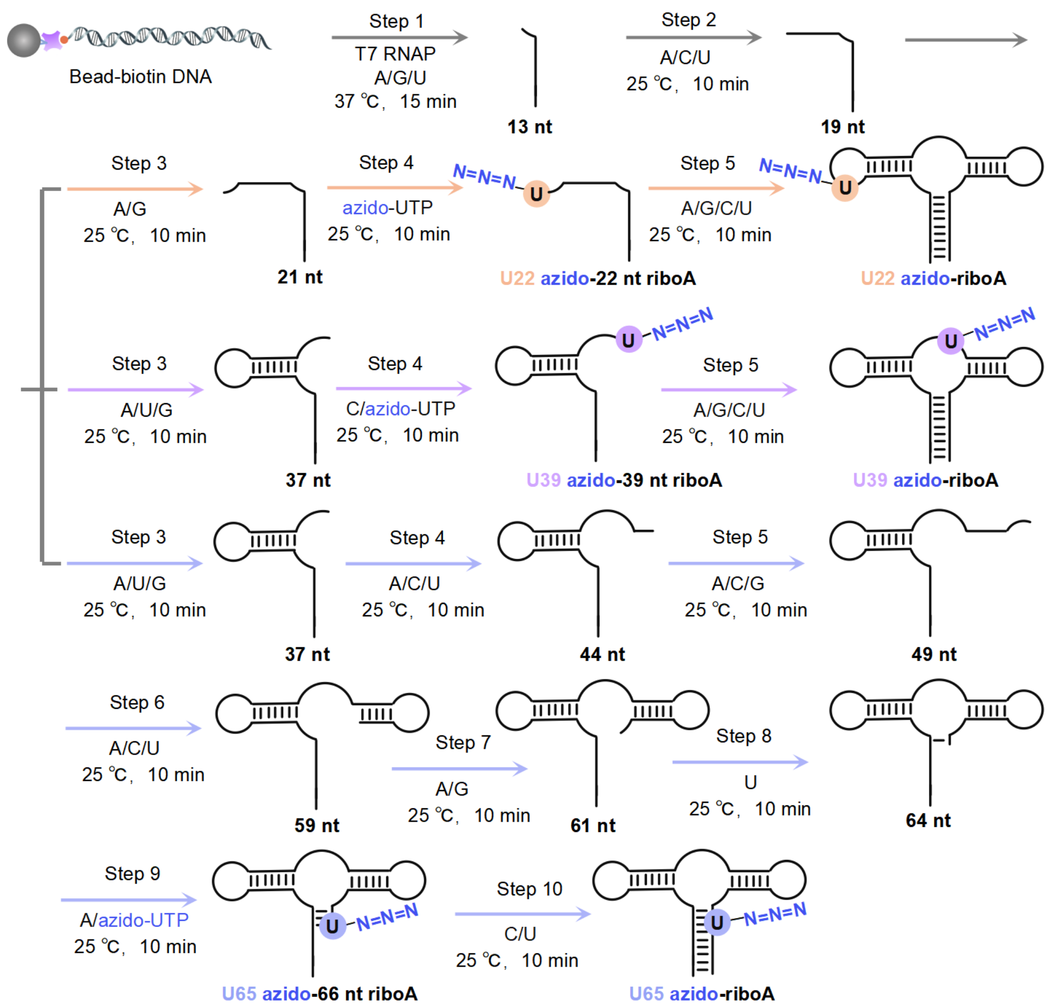

Supplementary Figure S9. The schematic procedures of 5-step, 5-step and 10-step PLOR for U22 azido-riboA, U39 azido-riboA and U65 azido-riboA synthesis, respectively.

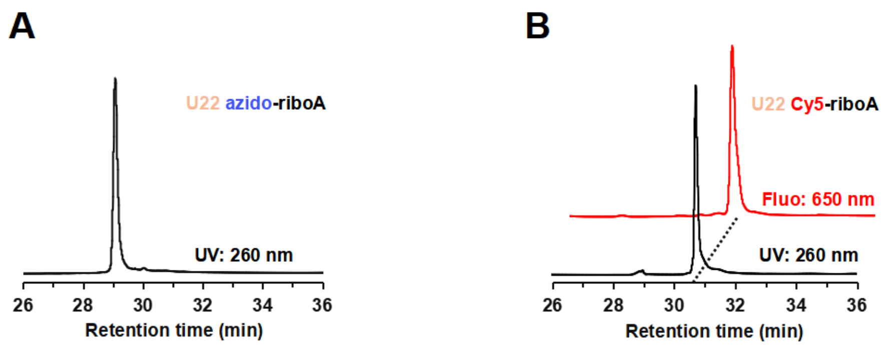

Supplementary Figure S10. Characterization of U22 Cy5-riboA via RP-HPLC. **(A)** HPLC spectrum of U22 azido-riboA at UV (260 nm) irridiation. **(B)** HPLC spectra of U22 Cy5-riboA under UV (260 nm, black line) and fluorescent (650 nm, red line) excitation.

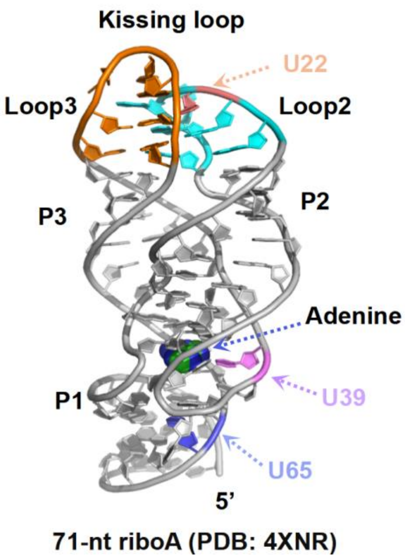

Supplementary Figure S11. The crystal structure of riboA. U22, U39, and U65 are shown in orange, purple and blue, respectively. The ligand, adenine is shown as colorful balls. Loop2 (shown in orange) and loop3 (shown in cyan) form the kissing loop, and U22 located near the kissing loop.

Supplementary Table S1. The DNA/RNA sequences used for riboSAM and riboA synthesis

DNA/RNA	Sequence
riboSAM	5′-GGGAAGAAAUAACAAUCAACGACGUAUCAUUGUGCCUCGCUUUCCGCGGGGCGAUAAGUCCUGAAGAAAGGGAUGAUAUGACAUAAGAACAUCGG
DNA template strand for riboSAM	5′-mCmCGATGTTCTTATGTCATATCATCCCTTTCTTCAGGACTTATCGCCCCGCGGAAAGCGAGGCACAATGATACGTCGTTGATTGTTATTTCTTCCCTATAGTGAGTCGTATTA*TGGACTAGCTGAATCAGA*
DNA coding strand for riboSAM	5′-biotin-*TCTGATTCAGCTAGTCCA*TAATACGACTCACTATAGGGAAGAAATAACAATCAACGACGTATCATTGTGCCTCGCTTTCCGCGGGGCGATAAGTCCTGAAGAAAGGGATGATATGACATAAGAACATCGG
Forward primer for riboSAM-DNA PCR	5’-biotin-TCTGATTCAGCTAGTCCATAATACGACT
Reverse primer for riboSAM-DNA PCR	5′-mCmCGATGTTCTTATGTCATATCATCCCTT
riboA	GGGAAGAUAUAAUCCUAAUGAUAUGGUUUGGGAGUUUCUACCAAGAGCCUUAAACUCUUGAUUAUCUUCCC
DNA template strand for riboA	5’-mGmGGAAGATAATCAAGAGTTTAAGGCTCTTGGTAGAAACTCCCAAACCATATCATTAGGATTATATCTTCCCTATAGTGAGTCGTATTA*TGGACTAGCTGAATCAGA*
DNA coding strand for riboA	5’-biotin-*TCTGATTCAGCTAGTCCA*TAATACGACTCACTATAGGGAAGATATAATCCTAATGATATGGTTTGGGAGTTTCTACCAAGAGCCTTAAACTCTTGATTATCTTCCC
Forward primer for riboA-DNA PCR	5’-biotin-TCTGATTCAGCTAGTCCATAATACGACT
Reverse primer for riboA-DNA PCR	5’-mGmGGAAGATAATCAAGAGTTTAAGGCTCT

The T7 promoter sequences in DNAs are underlined, and the linker sequences (italic) are inserted in DNA to alleviate potential steric hindrance between beads and T7 RNAP during transcription. mC/mG represents 2′-O-methyl-dC/dG.

Supplementary Table S2. Reagents usage in 7 step-PLOR to generate U43 azido-riboSAM and U43 Cy3-riboSAM

**Reagent usage (10 μM, 1 mL)**
**Step 1** in the buffer (6 mM $MgSO_4$, 40 mM Tris-HCl, 100 mM K_2SO_4, 10 mM DTT, pH 8.0) at 37 ºC for 15 min:<br>10 μM DNA-beads, 10 μM T7 RNAP, 1.12 mM ATP, 0.96 mM GTP and 32 μM UTP;<br>**Steps 2–7** in the buffer (6 mM $MgSO_4$, 40 mM Tris-HCl, 10 mM DTT, pH 8.0) at 25 ºC for 10 min except Step 6B at 30 ºC:<br>**Step 2**: 40 μM ATP, 30 μM CTP, 10 μM UTP;<br>**Step 3**: 10 μM ATP, 10 μM CTP, 20 μM GTP;<br>**Step 4**: 20 μM ATP, 10 μM CTP, 40 μM UTP;<br>**Step 5**: 30 μM GTP, 40 μM CTP, 20 μM UTP;<br>**Step 6A for U43 azido-riboSAM**: 20 μM ATP, 20 μM CTP, 10 μM azido-UTP ;<br>**Step 6B for U43 Cy3-riboSAM**: 20 μM ATP, 20 μM CTP, 10 μM Cy3-UTP ;<br>**Step 7**: 180 μM ATP, 70 μM CTP, 170 μM GTP, 80 μM UTP.

Supplementary Table S3. Reagents usage in 9 step-PLOR to generate U55 azido-riboSAM and U55 Cy3-riboSAM

**Reagent usage (10 μM, 1 mL)**
**Step 1** in the buffer (6 mM $MgSO_4$, 40 mM Tris-HCl, 100 mM K_2SO_4, 10 mM DTT, pH 8.0) at 37 ºC for 15 min:<br>10 μM DNA-beads, 10 μM T7 RNAP, 1.12 mM ATP, 0.96 mM GTP and 32 μM UTP;<br>**Steps 2–9** in the buffer (6 mM $MgSO_4$, 40 mM Tris-HCl, 10 mM DTT, pH 8.0) at 25 ºC for 10 min except Step 8B at 30 ºC:<br>**Step 2**: 40 μM ATP, 30 μM CTP, 10 μM UTP;<br>**Step 3**: 10 μM ATP, 10 μM CTP, 20 μM GTP;<br>**Step 4**: 20 μM ATP, 10 μM CTP, 40 μM UTP;<br>**Step 5**: 30 μM GTP, 40 μM CTP, 20 μM UTP;<br>**Step 6**: 20 μM ATP, 20 μM CTP, 10 μM UTP ;<br>**Step 7**: 20 μM CTP, 60 μM GTP ;<br>**Step 8A for U43 azido-riboSAM**: 30 μM ATP, 10 μM azido-UTP ;<br>**Step 8B for U43 Cy3-riboSAM**: 30 μM ATP, 10 μM Cy3-UTP ;<br>**Step 9**: 150 μM ATP, 50 μM CTP, 110 μM GTP, 70 μM UTP.

Supplementary Table S4. Reagents usage in 12 step-PLOR to generate U74 azido-riboSAM and U74 Cy3-riboSAM

Reagent usage (10 µM, 1 mL)
**Step 1** in the buffer (6 mM $MgSO_4$, 40 mM Tris-HCl, 100 mM K_2SO_4, 10 mM DTT, pH 8.0) at 37 ºC for 15 min:<br>10 µM DNA-beads, 10 µM T7 RNAP, 1.12 mM ATP, 0.96 mM GTP and 32 µM UTP;<br>**Steps 2–12** in the buffer (6 mM $MgSO_4$, 40 mM Tris-HCl, 10 mM DTT, pH 8.0) at 25 ºC for 10 min except Step 11B at 30 ºC:<br>**Step 2**: 40 µM ATP, 30 µM CTP, 10 µM UTP;<br>**Step 3**: 10 µM ATP, 10 µM CTP, 20 µM GTP;<br>**Step 4**: 20 µM ATP, 10 µM CTP, 40 µM UTP;<br>**Step 5**: 30 µM GTP, 40 µM CTP, 20 µM UTP;<br>**Step 6**: 20 µM ATP, 20 µM CTP, 10 µM UTP ;<br>**Step 7**: 20 µM CTP, 60 µM GTP ;<br>**Step 8**: 30 µM ATP, 10 µM GTP, 20 µM UTP ;<br>**Step 9**: 20 µM CTP, 10 µM GTP, 10 µM UTP ;<br>**Step 10**: 60 µM ATP, 40 µM GTP ;<br>**Step 11A for U74 azido-riboSAM**: 10 µM GTP, 10 µM azido-UTP ;<br>**Step 11B for U74 Cy3-riboSAM**: 10 µM GTP, 10 µM Cy3-UTP ;<br>**Step 12**: 90 µM ATP, 30 µM CTP, 40 µM GTP, 40 µM UTP.

Supplementary Table S5. Reagents usage in 12 step-PLOR to generate U84 azido-riboSAM and U84 Cy3-riboSAM

**Reagent usage (10 μM, 1 mL)**
**Step 1** in the buffer (6 mM $MgSO_4$, 40 mM Tris-HCl, 100 mM K_2SO_4, 10 mM DTT, pH 8.0) at 37 ºC for 15 min:<br>10 μM DNA-beads, 10 μM T7 RNAP, 1.12 mM ATP, 0.96 mM GTP and 32 μM UTP;<br>**Steps 2–12** in the buffer (6 mM $MgSO_4$, 40 mM Tris-HCl, 10 mM DTT, pH 8.0) at 25 ºC for 10 min except Step 11B at 30 ºC:<br>**Step 2**: 40 μM ATP, 30 μM CTP, 10 μM UTP;<br>**Step 3**: 10 μM ATP, 10 μM CTP, 20 μM GTP;<br>**Step 4**: 20 μM ATP, 10 μM CTP, 40 μM UTP;<br>**Step 5**: 30 μM GTP, 40 μM CTP, 20 μM UTP;<br>**Step 6**: 20 μM ATP, 20 μM CTP, 10 μM UTP ;<br>**Step 7**: 20 μM CTP, 60 μM GTP;<br>**Step 8**: 30 μM ATP, 10 μM GTP, 20 μM UTP ;<br>**Step 9**: 20 μM CTP, 10 μM GTP, 10 μM UTP ;<br>**Step 10**: 90 μM ATP, 60 μM GTP, 30 μM UTP ;<br>**Step 11A for U74 azido-riboSAM**: 40 μM ATP, 10 μM CTP, 10 μM azido-UTP ;<br>**Step 11B for U74 Cy3-riboSAM**: 40 μM ATP, 10 μM CTP, 10 μM Cy3-UTP ;<br>**Step 12**: 30 μM ATP, 20 μM CTP, 30 μM GTP, 10 μM UTP.

Supplementary Table S6. Reagents usage in 5 step-PLOR to generate U22 azido-riboA

**Reagent usage (15 μM, 1 mL)**
**Step 1** in the buffer (6 mM $MgSO_4$, 40 mM Tris-HCl, 100 mM K_2SO_4, 10 mM DTT, pH 8.0) at 37 ºC for 15 min:<br>15 μM DNA-beads, 15 μM T7 RNAP, 1.44 mM ATP, 0.96 mM GTP and 144 μM UTP;<br>**Steps 2–5** in the buffer (6 mM $MgSO_4$, 40 mM Tris-HCl, 10 mM DTT, pH 8.0) at 25 ºC for 10 min:<br>**Step 2**: 30 μM ATP, 30 μM CTP, 30 μM UTP;<br>**Step 3**: 15 μM ATP, 15 μM GTP;<br>**Step 4**: 15 μM azido-UTP;<br>**Step 5**: 165 μM ATP, 165 μM CTP, 120 μM GTP, 285 μM UTP.

Supplementary Table S7. Reagents usage in 5 step-PLOR to generate U39 azido-riboA

**Reagent usage (15 µM, 1 mL)**
**Step 1** in the buffer (6 mM $MgSO_4$, 40 mM Tris-HCl, 100 mM K_2SO_4, 10 mM DTT, pH 8.0) at 37 ºC for 15 min:<br>15 µM DNA-beads, 15 µM T7 RNAP, 1.44 mM ATP, 0.96 mM GTP and 144 µM UTP;<br>**Steps 2–5** in the buffer (6 mM $MgSO_4$, 40 mM Tris-HCl, 10 mM DTT, pH 8.0) at 25 ºC for 10 min:<br>**Step 2**: 30 µM ATP, 30 µM CTP, 30 µM UTP;<br>**Step 3**: 45 µM ATP, 105 µM GTP, 120 µM UTP;<br>**Step 4**: 15 µM CTP, 15 µM azido-UTP;<br>**Step 5**: 135 µM ATP, 150 µM CTP, 30 µM GTP, 165 µM UTP.

Supplementary Table S8. Reagents usage in 10 step-PLOR to generate U65 azido-riboA

**Reagent usage (15 μM, 1 mL)**
**Step 1** in the buffer (6 mM $MgSO_4$, 40 mM Tris-HCl, 100 mM K_2SO_4, 10 mM DTT, pH 8.0) at 37 ºC for 15 min:<br>15 μM DNA-beads, 15 μM T7 RNAP, 1.44 mM ATP, 0.96 mM GTP and 144 μM UTP;<br>**Steps 2–10** in the buffer (6 mM $MgSO_4$, 40 mM Tris-HCl, 10 mM DTT, pH 8.0) at 25 ºC for 10 min:<br>**Step 2**: 30 μM ATP, 30 μM CTP, 30 μM UTP;<br>**Step 3**: 45 μM ATP, 105 μM GTP, 120 μM UTP;<br>**Step 4**: 45 μM ATP, 45 μM CTP, 15 μM UTP;<br>**Step 5**: 15 μM ATP, 30 μM CTP, 30 μM GTP;<br>**Step 6**: 45 μM ATP, 30 μM CTP, 75 μM UTP;<br>**Step 7**: 15 μM ATP, 15 μM GTP;<br>**Step 8**: 45 μM UTP;<br>**Step 9**: 15 μM ATP, 15 μM azido-UTP;<br>**Step 10**: 60 μM CTP, 30 μM UTP.
